# *Aedes albopictus* arrives in Lisbon: an emerging public health threat

**DOI:** 10.3389/fpubh.2023.1332334

**Published:** 2023-12-19

**Authors:** Teresa Nazareth, Gonçalo Seixas, José Lourenço, Paulo J. G. Bettencourt

**Affiliations:** ^1^Faculty of Medicine, Universidade Católica Portuguesa, Rio de Mouro, Portugal; ^2^Center for Interdisciplinary Research in Health, Universidade Católica Portuguesa, Rio de Mouro, Portugal; ^3^Global Health and Tropical Medicine (GHTM), Associate Laboratory in Translation and Innovation Towards Global Health, LA-REAL, Instituto de Higiene e Medicina Tropical, Universidade Nova de Lisboa (IHMT/UNL), Lisbon, Portugal; ^4^Católica Biomedical Research Center, Faculty of Medicine, Universidade Católica Portuguesa, Rio de Mouro, Portugal; ^5^Climate Amplified Diseases and Epidemics (CLIMADE), Lisbon, Portugal

**Keywords:** *Aedes albopictus*, dengue, Zika, chikungunya, yellow fever, global expansion of mosquito-borne diseases, climatic change, mRNA vaccines

## Introduction

The recent identification in Lisbon, Portugal, of the invasive mosquito species *Aedes albopictus (Ae. albopictus)*, also known as the Asian tiger mosquito, was recently reported by the Public Health authorities (1). This raised significant concerns among the local scientific community, even though viruses were not identified within these mosquitoes. Originating from Southeast Asia, the species is a major vector for several arboviruses, including dengue, Zika, chikungunya, and yellow fever (2), and its increasing presence in areas beyond the classic geographical range underscores concern regarding the potential global expansion of mosquito-borne diseases.

For some decades, *Aedes* species' distribution was thought to be restricted to equatorial, subequatorial, and tropical zones (corresponding to the sub-Saharan Africa, Southeast Asia, and part of the South and Central America). However, in recent years, these mosquitoes have been able to occupy areas above and below these isotherms eventually also reaching temperate zones (which include Europe and North America, but also South America e.g., Argentina and Chile).

## The invasive journey of *Aedes spp* into Europe and North America

*Aedes albopictus* has been on a global invasive journey toward Europe and North America since the 1980s. First reported in Albania in 1979, and later in Texas (USA) in 1985, it established populations in several European countries and spread to more than 32 states in the USA (3, 4). In recent years it was implicated in short-term outbreaks of chikungunya and dengue in Italy, France, Florida, Texas, and Arizona (5, 6).

*Aedes albopictus* eggs can stand extended periods of desiccation resisting even in extreme conditions through diapausing mechanisms. Facilitated by human activities, *Ae. albopictus* eggs are capable of dispersing over considerable distances in short periods of time, facilitating the spread into new territories, provided there are favorable ecosystems for survival and reproduction. Like any other mosquito, *Ae. albopictus* requires water bodies to lay eggs that will hatch into larvae and develop to pupae before emerging as adult mosquitoes. *Ae. albopictus*' ability to breed in small amounts of water, and its evolutive preference by artificial water containers, commonly found in urban areas, facilitates the proliferation of the mosquito populations and its rapid expansion in densely populated regions. Usually, these water containers that act as breeding sites are part of dwellings turning the local human community into a critical stakeholder in vector control strategies.

In addition to its ecological plasticity, *Ae. albopictus* benefits from human economical activities such as the frequent exchange of goods and travelers, facilitated by globalization and further supported by climate change (2, 3). Moreover, if the mosquito population establishes in a new region, and survives throughout the winter season, eradication becomes very challenging (7).

Historical records suggest the presence of an *Aedes* species in North America and Europe prior to World War II, which may have been eradicated during the eradication of malaria *Anopheles* vectors through Dichlorodiphenyltrichloroethane (DDT) applications, a widely used insecticide in vector control campaigns (8, 9). Currently DDT is not used due to well-known risks for human health and the environment, as well mosquito populations showing high levels of resistance to DDT and other common insecticides (10).

Stringent surveillance and sustainable control of *Ae. albopictus* needs to be implemented by Public Health authorities to stop the introduction of associated mosquito-borne viruses, but so far no consensus exists on how this can be achieved.

*Aedes aegypti*, a mosquito species closely related to *Ae. albopictus*, is also expanding its range, particularly in North America. While it demonstrates less ecological plasticity, *Ae. aegypti* is often considered a more efficient vector for viral transmission, being a primary vector of several arboviruses, including dengue, Zika, chikungunya, and yellow fever. In 2005, *Ae. aegypti* was identified on Island of Madeira, Portugal. Subsequently, in 2012, the island faced the first dengue outbreak with +2,000 human confirmed cases (11). During the last two decades, Portugal's mainland escaped the importation of this species but *Ae. albopictus is now regularly observed*, most likely introduced from neighboring Mediterranean countries. Its presence in Portugal was first detected in 2017, with populations recorded in the North of the country, and the Algarve and Alentejo districts (South) (1, 12). Its recent identification in September 2023 in Lisbon, is a warning for the urgent need for stringent surveillance and Public Health control interventions. Portugal can benefit of the expertise and knowledge acquired through prior work done in Madeira and develop a strategy based on tailored communication initiatives, to promote effective community-based prevention (13).

## Discussion

The continuous invasion of *Ae. albopictus* represents an increasing threat of yellow fever, dengue, Zika, and chikungunya, to immunologically naïve populations, with the risk of expansion at a global scale (14). The changing distribution of this vector highlights the need for heightened awareness and action in both the Global North and the Global South, which is crucial for a unified global response against infectious diseases. Addressing these public health challenges particularly by improving surveillance, establishing efficient control measures, and preparedness for the development of new vaccines against known pathogens in immunological naïve populations, particularly against Zika and chikungunya due to the absence of licensed vaccines. The recent success of mRNA vaccines created a wide interest for the rapid development of vaccines against virtually every infectious disease, and Zika and chikungunya mRNA vaccine candidates are currently in Phase I clinical trials (15).

Climatic change is playing an important role, facilitating the establishment of vectors and viruses beyond their classic tropical and subtropical geographical boundaries, by favoring e.g., environmental conditions for mosquito reproduction. While this presents a challenge into the future, it also provides opportunities for research, surveillance and control. Natural climate variation is such a determinant factor to mosquito life cycles (16–18), that it is a powerfully informative input to emerging mathematical modeling techniques capable of projecting the dynamics and spatial distributions of both mosquitoes and viruses (19). The contribution of this field of research into the future is ever more relevant, since it can pinpoint spatial-temporal details for optimized surveillance and control, which are needed to effectively stop the establishment of *Aedes spp* into new territories.

Although *Ae. albopictus* has been identified in several regions of Portugal, it is the first time to be identified in Lisbon. The latter is the largest urban center in the country and a hub of international travel including frequent movement to/from South America and Southeast Asia where arboviruses of public health concern are endemic. This serves as a pressing reminder and a call to action for the scientific community, Public Health authorities and civil society, emphasizing the emerging threat posed by invasive mosquito species and the diseases they amplify. Collaborative and proactive efforts, including extensive Public Engagement are essential to implement effective control measures, raise awareness, and protect communities from the spread of this invasive vector. The time to act is now, investing in communication, surveillance and research, toward preventing the establishment and proliferation of *Ae. albopictus* in new national territories, to protect our communities from eventual forthcoming disease outbreaks.

## Author contributions

TN: Conceptualization, Writing—original draft, Writing—review & editing. GS: Writing—original draft, Writing—review & editing. JL: Writing—original draft, Writing—review & editing. PB: Conceptualization, Writing—original draft, Writing—review & editing.

## References

[1] Relatório de Resposta sazonal em saúde - Vigilância e monitorização. (2023). Lisboa. Available online at: https://www.dgs.pt/ficheiros-de-upload-2013/relatorio-n-43-da-resposta-sazonal-em-saude-vigilancia-e-monitorizacao-pdf.aspx (accessed October 30, 2023).

[2] Bonizzoni M Gasperi G Chen X James AA. The invasive mosquito species Aedes albopictus: current knowledge and future perspectives. Trends Parasitol. (2013) 29:460–8. 10.1016/j.pt.2013.07.003 23916878 PMC3777778

[3] Kraemer MUG Sinka ME Duda KA Mylne AQN Shearer FM Barker CM. The global distribution of the arbovirus vectors Aedes aegypti and Ae. albopictus. Elife. (2015) 4:e08347. 10.7554/eLife.08347 26126267 PMC4493616

[4] Medlock JM Hansford KM Schaffner F Versteirt V Hendrickx G Zeller H. A review of the invasive mosquitoes in Europe: ecology, public health risks, and control options. Vector Borne Zoonotic Dis. (2012) 12:435–47. 10.1089/vbz.2011.0814 22448724 PMC3366101

[5] Cassaniti I Ferrari G Senatore S Rossetti E Defilippo F Maffeo M. Preliminary results on an autochthonous dengue outbreak in Lombardy Region, Italy, August 2023. Euro Surveill. (2023) 28:2300471. 10.2807/1560-7917.ES.2023.28.37.2300471 37707980 PMC10687988

[6] Cochet A Calba C Jourdain F Grard G Durand GA Guinard A. Autochthonous dengue in mainland France, 2022: geographical extension and incidence increase. Euro Surveill. (2022) 27:2200818. 10.2807/1560-7917.ES.2022.27.44.2200818 36330819 PMC9635021

[7] Paupy C Delatte H Bagny L Corbel V Fontenille D. Aedes albopictus, an arbovirus vector: from the darkness to the light. Microbes Infect. (2009) 11:1177–85. 10.1016/j.micinf.2009.05.005 19450706

[8] Gubler DJ. The changing epidemiology of yellow fever and dengue, 1900 to 2003: full circle? Comp Immunol Microbiol Infect Dis. (2004) 27:319–30. 10.1016/j.cimid.2004.03.013 15225982

[9] Sadasivaiah S Tozan Y Breman JG. Dichlorodiphenyltrichloroethane (DDT) for indoor residual spraying in Africa: how can it be used for malaria control? Am J Trop Med Hyg. (2007) 77(6 Suppl):249–63. 10.4269/ajtmh.2007.77.249 18165500

[10] Bettencourt P. Current challenges in the identification of pre-erythrocytic malaria vaccine candidate antigens. Front Immunol. (2020) 11:190. 10.3389/fimmu.2020.00190 32153565 PMC7046804

[11] Sousa CA Clairouin M Seixas G Viveiros B Novo MT Silva AC. Ongoing outbreak of dengue type 1 in the autonomous region of madeira, Portugal: preliminary report. Euro Surveill. (2012) 17:20333. 10.2807/ese.17.49.20333-en 23231893

[12] Osório HC Zé-Zé L Neto M Silva S Marques F Silva AS. Detection of the invasive mosquito species aedes (Stegomyia) albopictus (diptera: Culicidae) in Portugal. Int J Environ Res Public Health. (2018) 15:820. 10.3390/ijerph15040820 29690531 PMC5923862

[13] Nazareth T Teodósio R Porto G Gonçalves L Seixas G Silva AC. Strengthening the perception-assessment tools for dengue prevention: a cross-sectional survey in a temperate region Madeira, Portugal. BMC Public Health. (2014) 14:39. 10.1186/1471-2458-14-39 24428823 PMC3905660

[14] Nie P Feng J. Niche and range shifts of *Aedes aegypti* and *Ae. albopictus* suggest that the latecomer shows a greater invasiveness. Insects. (2023) 14:810. 10.3390/insects14100810 37887822 PMC10607146

[15] Matarazzo L Bettencourt PJG. mRNA vaccines: a new opportunity for malaria, tuberculosis and HIV. Front Immunol. (2023) 14:1172691. 10.3389/fimmu.2023.1172691 37168860 PMC10166207

[16] Ryan SJ Carlson CJ Mordecai EA Johnson LR. Global expansion and redistribution of Aedes-borne virus transmission risk with climate change. PLoS Negl Trop Dis. (2018) 13:e0007213. 10.1371/journal.pntd.0007213 30921321 PMC6438455

[17] Dostal T Meisner J Munaycoid C García PJ Cárcamo C Lu JEP. The effect of weather and climate on dengue outbreak risk in Peru, 2000-2018: a time-series analysis. PLoS Negl Trop Dis. (2022) 16:e0010479. 10.1371/journal.pntd.0010479 35771874 PMC9278784

[18] Piovezan-Borges AC Valente-Neto F Urbieta GL Laurence SGW de Oliveira Roque F. Global trends in research on the effects of climate change on Aedes aegypti: international collaboration has increased, but some critical countries lag behind. Parasit Vectors. (2022) 15:346. 10.1186/s13071-022-05473-7 36175962 PMC9520940

[19] Nakase T Giovanetti M Obolski U Lourenço J. Global transmission suitability maps for dengue virus transmitted by Aedes aegypti from 1981 to 2019. Sci Data. (2023) 10:275. 10.1038/s41597-023-02170-7 37173303 PMC10182074

